# Extending the Transformative Potential of Mindfulness Through Team Mindfulness Training, Integrating Individual With Collective Mindfulness, in a High-Stress Military Setting

**DOI:** 10.3389/fpsyg.2022.867110

**Published:** 2022-06-30

**Authors:** Jutta Tobias Mortlock, Alison Carter, Dawn Querstret

**Affiliations:** ^1^Department of Psychology, City University of London, London, United Kingdom; ^2^Centre for Business Performance, Cranfield University School of Management, Cranfield, United Kingdom; ^3^Institute for Employment Studies, Brighton, United Kingdom; ^4^Department of Psychology and Pedagogic Science, St Mary’s University, London, United Kingdom

**Keywords:** mindfulness, collective mindfulness, mindful organizing, mindfulness interventions, military, randomized controlled trial, stress management, meditation

## Abstract

Mindfulness has come to be considered an important approach to help individuals cultivate transformative capacity to free themselves from stress and suffering. However, the transformative potential of mindfulness extends beyond individual stress management. This study contributes to a broadening of the scope of contemplative science by integrating the prominent, individually focused mindfulness meditation literature with collective mindfulness scholarship. In so doing, it aims to illuminate an important context in which mindfulness interventions are increasingly prevalent: workplaces. Typically, the intended effect of workplace mindfulness training is to help workers manage stress better. Since mindfulness in organizations impacts individual and collective processes, the study blends the above literatures to create a cross-level “next-generation” Team Mindfulness Training (TMT) pilot. Its potential in helping individuals and teams to manage work stress better is investigated *via* a two-phase mixed-methods research study in high-stress military work populations, and compared to a conventional (“first-generation”) 8-week mindfulness meditation program based on mindfulness-based stress reduction (MBSR). Results suggest that compared to the “first-generation” mindfulness program, TMT seems no less effective in raising individual stress management skills, and may hold more promise in generating collective capacity to manage stress and unexpected difficulty, linked to an apparent interdependence between collective and individual mindfulness capacity development. Based on these empirical results, the study contributes to theory in three important ways: first, it outlines how individual and collective mindfulness in workplaces may be interdependent. Second, it explains why “next-generation” workplace training interventions should apply a cross-level approach. And third, it illustrates how its transformative potential for people at work, individually as well as collectively, can be extended by moving beyond an inward-looking meditation focus in mindfulness training. The study contributes to practice by providing a detailed outline of the pilot TMT program, and offers a series of follow-up research opportunities to inspire further scientific innovation in workplace mindfulness training, especially for high-stress work populations. The study’s ultimate aim is to prompt a shift away from adapting clinically oriented, self-focused “first-generation” mindfulness training protocols, and towards *mindfulness as team sport*: a more prosocially oriented mindfulness science intent on generating wisdom and compassion, for one and all.

## Introduction

### Research Background

Mindfulness is a cognitive process (Vago and Silbersweig, 2012; Lutz et al., 2015) available to humanity to develop particular skills, centered around present-moment awareness and acceptance (Lindsay and Creswell, 2017). These skills often describe the *what* of mindfulness—paying attention to present-moment experience—and the *how*—with an open-minded, accepting attitude (Kabat-Zinn, 1994; Eisenlohr-Moul et al., 2012). The *why* of mindfulness may be even more important and is at the heart of the present inquiry. Mindfulness can be considered an important approach to cultivating transformative inner qualities “in the service of wisdom in difficult circumstances” (Kabat-Zinn, 2011, p. 5). Wisdom in this context helps individuals on one hand *understand* the nature of suffering and conflicts of interest, not only in their minds but also in the world. On the other, it generates transformative capacity to *release* human suffering and peacefully resolve intrapsychic as well as interpersonal conflicts.

Most mindfulness-based interventions (MBIs) today originate from the seminal mindfulness-based stress reduction (MBSR) program created by Jon Kabat-Zinn in the 1970s (Kabat-Zinn, 1982, 1994; Creswell, 2017). MBSR is one of a potentially infinite number of “skillful means” to generate wisdom and healing from suffering (Kabat-Zinn, 2011, p. 3). MBIs were originally designed as “participatory medicine,” bringing together contemplative traditions and clinical medical science, to help hospital patients suffering clinical or mental health conditions find relief (*ibid.*). MBIs predominately use meditation—which can be defined operationally as self-regulation of attention (Goleman and Schwartz, 1976)—to cultivate wise, transformative awareness, so that it might become possible for people to take care of their wellbeing *by themselves*, in addition to receiving treatment for their medical conditions (Kabat-Zinn, 2011). This skillful way of being and encountering difficulty is, in today’s everyday language, about managing stress. In simple terms, mindfulness training inspired by MBSR helps people develop valuable individual stress management skills.

Kabat-Zinn’s seminal work spawned the so-called first-generation of MBIs, rooted in Buddhist traditions and designed for secular community settings in order to be “maximally accessible to people with diverse values and religious affiliations” (Crane et al., 2017, p. 991).

Over the last 30 years, psychology scholars and behavioral medicine experts have introduced MBIs to a variety of different contexts beyond hospitals (Creswell, 2017). MBIs have been extensively researched and their effectiveness in generating individual stress management capability is broadly confirmed. Systematic reviews and meta-analyses with clinical samples have demonstrated that MBIs are effective in reducing depression (Kuyken et al., 2015; Goldberg et al., 2018). Meta-analyses of MBIs with non-clinical samples indicate significant stress reduction (Chiesa and Serretti, 2009), increased well-being (Eberth and Sedlmeier, 2012), and higher quality of life (Khoury et al., 2015).

However, the transformative potential of mindfulness stretches beyond individual stress management. While “first-generation” MBIs focus on inner transformation and serve predominately as a clinical tool, a self-help tool, and a mental training tool (Crane et al., 2017), recall that the ultimate vision for MBSR included wisdom to relieve suffering and resolve conflicts in the face of difficulty not only *within* but also *between* individuals and in the world at large. This means mindfulness may be not only helpful for *individual* stress management but also for *collective* stress management.

### Study Focus

Building on the conceptualization of Crane et al. (2017), this study explores “next-generation” theorizing on mindfulness training, in particular a shift away from MBIs serving as self-help tool and towards a social catalyst for transformation. Its focus is on illuminating an important context in which mindfulness interventions are increasingly prominent: workplaces.

More specifically, the paper seeks to build bridges across the individually-focused mindfulness meditation literature centered around MBSR and towards another mindfulness literature that is less intrapsychic in orientation and instead more focused on social and situational awareness: the collective mindfulness literature dedicated to studying team processes of shared cognition and action that help teams and entire organizations uncover and overcome unexpected stressors and thus “manage the unexpected” (Weick et al., 1999).

In contrast to a focus on *individual* stress management through MBSR and related mindfulness training programs, collective mindfulness is a social construct, defined as a group’s capability to notice significant issues and emergent errors in situations and to jointly act on what they observe (Weick et al., 2000). In other words, employees acting mindfully on a collective scale manage stress *collectively*: they are able to anticipate, detect, and appropriately respond to unexpected, stressful problems (Weick et al., 1999; Weick and Sutcliffe, 2007).

Collective mindfulness arises out of specific social practices, actions, and communication patterns that liken the “collective mind” of a group of individuals who organize mindfully to a flock of birds flying in unison, with each bird constantly paying attention not only to their own direction, but also to every other member of the flock, and constantly aligning individual action with the overall direction of the collective (Weick and Roberts, 1993).

Because collective mindfulness is enacted through a dynamic process of social action and interaction, it is also referred to as *mindful organizing* (MO; Sutcliffe et al., 2016), to emphasize its non-static, ever-evolving nature. Originally, the concept of MO was developed to explain how High-Reliability Organizations (HROs) develop capacity to avoid catastrophic failure and perform in nearly error-free ways despite operating in extreme, stressful conditions; however, its scope has expanded to also apply to teams and organizations that are capable of being aware of the status quo in order to improve it, refusing to operate on “auto pilot” (Fiol and O’Connor, 2003; Sutcliffe et al., 2016).

While MO may appear to align closely with standard management practice, Weick et al. emphasize that interpersonal skills in HROs are as important as technical skills (Weick et al., 1999). Teams who organize mindfully “are motivated to work for the benefit of others and are more receptive to others’ perspectives and incorporate those perspectives into their work” (Vogus et al., 2014, p. 592): collective mind in action. The origin of this interpersonal mindset stems from prosociality; attitudes and behaviors intended to benefit others (Batson and Powell, 2003), and the capacity to be emotionally ambivalent, i.e., capable of experiencing positive and negative emotions at the same time, for example feeling hope as well as doubt (Vogus et al., 2014). Five collective mindfulness processes generate MO: (1) Sensitivity to Operations; (2) Preoccupation with Failure; (3) Reluctance to Simplify; (4) Commitment to Resilience; and (5) Deference to Expertise (Weick et al., 2000).

In sum, the prosocial nature and other-orientedness of collective mindfulness render it pertinent for the endeavor of the present study and its ultimate aim: expand the potential for mindfulness training to make a positive difference in the world.

To date, the collective mindfulness scholarship has largely bypassed prominent mindfulness science debates. This is probably because collective mindfulness is rooted in management science, not a combination of contemplative and clinical science. In addition, it conflicts with the assertion that mindfulness can only be understood from the inside out, as an embodied practice and first-person experience of awareness that is not about intellect or cognition, and instead nurtured through the second-person perspective of a highly skilled trainer (Kabat-Zinn, 2011). Finally, to the best of the authors’ knowledge, no standard training protocol to generate collective mindfulness in organizations has been published in the peer-reviewed literature. This means that organizations interested in evidence-based methods to bring mindfulness to their employees tend to rely on “first-generation” MBIs, at least as a starting point for their learning and development initiatives.

### Why This Study Is Timely

The integration of the individually focused mindfulness research with collective mindfulness scholarship is timely, for at least four reasons beyond the afore-mentioned aspiration of MBSR to contribute to healing and wisdom in an ever-increasing range of mainstream settings.

First, other-orientedness is embedded in Buddhist heritage, yet the contemporary debate on mindfulness frequently overlooks an integration of “the by-now almost traditional road to personal wellbeing with other-regarding actions that arise from other-regarding motives” (Van Doesum et al., 2013). In fact, the Dalai Lama emphasizes in his teachings that Eastern contemplative traditions anchor around the link between mindfulness practice and an altruistic mindset (Dalai Lama and Ekman, 2008). When it comes to practicing mindfulness, the Dalai Lama explains, “the correct motivation is the altruistic attitude” (Dalai Lama, 2005, p. 168).

Second, while prominent mindfulness debates focus on mindfulness as an inner quality (Bishop et al., 2004; Kabat-Zinn, 2005), mindfulness in organizations is generally considered as a concept that stretches beyond individual-level considerations. In fact, workplace mindfulness is a cross-level construct, embedded in interpersonal relations and interactions, emerging through meditative as well as nonmeditative practices, by individuals as well as by groups of people interacting with each other *mindfully* (Sutcliffe et al., 2016). Moreover, mindfulness is not the same as meditation. While meditation is generally considered a primary method for becoming mindful, mindfulness can arise organically in everyday life (Reina and Kudesia, 2020). In addition, leading mindfulness scholars argue that conflating mindfulness with meditation alone may be a Western misreading of Buddhist philosophy, at the expense of using other forms of mindfulness practice to cultivate valuable qualities (Lopez, 2010), and warn that an exclusive intrapsychic focus of mindfulness practice is liable to reduce its relevance to people’s daily lives “off the cushion” (Brown and Ryan, 2004, p. 246). Indeed, the physical and mental health benefits of non-meditative mindfulness practices are well documented (Alexander et al., 1989; Carson and Langer, 2006; Haigh et al., 2011; Pagnini et al., 2015). This is relevant for debates on expanding the potential of mindfulness for workplaces because so far, fewer than one in seven United States adults have tried meditation (Clarke et al., 2018). If individuals or organizations are not interested in meditation for any reason, they should not be “doomed to a life of mindlessness” (Pagnini and Langer, 2015, p.365).

Third, this research responds to calls from mindfulness scholars urging a broader, more context-aware debate on how mindfulness may benefit people at work (Rupprecht et al., 2019) and for integrating more diverse schools of thought in workplace mindfulness research (Selart et al., 2020). The study integrates individual meditation-focused mindfulness practice, as spearheaded by MBSR and related intervention programs, with mindfulness practice that is interpersonal in nature, such as practicing compassionate attitudes and behavior, i.e., working for the benefit of other sentient beings (Dalai Lama, 2005), or managing stressful events collectively, as in mindful organizing (Weick and Sutcliffe, 2007). In so doing, the study aims to help address increasing scientific criticism of both of these seminal studies. For example, meditation-focused MBSR scholars warn that MBSR and programs deriving from it are seen as a “one-size-fits-all” approach (Van Dam et al., 2017), “essentially replicating clinical mindfulness research in the workplace” (Reb et al., 2020; p. 3), while MO scientists lament that the antecedents of collective mindfulness are poorly understood (Argote, 2006).

Finally, both are mature literatures, employing distinct research methodologies (MBSR and its derivatives draw largely on quantitative intervention studies while the collective mindfulness literature tends to focus on qualitative observational and case study work), yet few empirical integrations of these schools of thought exists to date (notable exceptions are Fraher et al., 2017; Yu and Zellmer-Bruhn, 2018; and Liu et al., 2020). This presents untapped opportunities for exploring synergies between them.

### The Study’s Research Approach

The present research approach is a multi-phase mixed-methods pilot study in a high-stress work population: military officers in training. Specifically, the study investigates the potential—in other words, potentially unrealized benefits—of a newly created “next-generation” mindfulness training intervention that combines essential features of “standard” individual mindfulness training with the hallmarks of collective mindfulness.

Two features of this newly created pilot intervention are important for explaining the underlying rationale for this study.

First, the intervention under study is named “Team Mindfulness Training (TMT),” leaning on the *team mindfulness* construct, a relatively recent mindfulness construct (Yu and Zellmer-Bruhn, 2018). This is because team mindfulness sits at an interpersonal level of analysis and is thus a meso-level construct between the individual-level focus often applied to individual mindfulness, and the organization-level lens typically applied to collective mindfulness processes. Team mindfulness is defined as a team’s shared belief that team members’ interactions are marked by attention and awareness of the present moment as well as nonjudgment in the space between individual team members, and this shared belief reduces conflict and antisocial team behavior such as interpersonal undermining of other team members (Yu and Zellmer-Bruhn, 2018). While Yu and Zellmer-Bruhn (*ibid.*) do not specify what types of interventions generate team mindfulness, they nonetheless argue that it represents a promising approach for team conflict reduction. The present work extends this scholarship by providing a comprehensive outline of a pilot TMT intervention program, targeting both individual and collective (interpersonal) mindfulness practices and processes, in order to advance understanding of how to generate team mindfulness and thus create mental space both *within* individuals and *in the space between* them.

Second, this work is aligned with Kudesia (2019) who argues that in order to understand the potential of mindfulness for workplaces scholars need a mindset shift from “borrowing,” or adapting, extant mindfulness training approaches (as is the norm currently for workplace MBIs) towards “blending” concepts and ideas (Oswick et al., 2011; cited in Kudesia, 2019). Considering mindfulness *as team sport* does just that.

The research question (RQ) guiding this inquiry is:

*Can TMT generate individual and collective stress management skills, and if so, how?*

This paper is structured as follows:

First, the study’s methodology is presented, including a detailed overview of the pilot cross-level TMT intervention for workplaces. Then, Study 1 explores the general potential of TMT, while Study 2 compares its potential to a slight adaptation of “traditional” MBSR. This is followed by a presentation of the study’s findings. The paper concludes with a discussion of the findings in relation to theory-building and by generating recommendations for follow-up research and practice.

## Materials and Methods

### Methodology Overview

The research methodology consisted of Study 1, a pre-pilot intervention of the TMT program with a small sample of junior military personnel (*n* = 23) from a British Army military training division, evaluated qualitatively (*n* = 21). This was followed by Study 2, a mixed method controlled pilot intervention with a second set of junior military personnel from four Royal Navy military training divisions (divisions are administrative units serving as work teams). A sample of 105 individuals participated in the intervention; for full details on participation in its evaluation please see Supplementary Material. In Study 2, participation in the TMT program was compared to participation in an MBSR intervention minimally adapted for a military context.

The quantitative part of Study 2 comprised a block randomized controlled comparison of TMT with MBSR (allocating participants to condition *en bloc* by the division to which they belonged). It followed a 2 × 3 between subjects repeated measures design, comparing TMT to MBSR (which served as active control condition). Outcome variables were self-reported perceptions of individual resilience (Campbell-Sills and Stein, 2007) and perceptions of mindful organizing (Vogus and Sutcliffe, 2007b) in participant divisions, administered using pen and paper. In addition, a computer-based working memory test (Unsworth et al., 2005) was used, serving as proxy for objectively measuring individual performance under pressure. These assessments were taken at three times: at baseline, i.e., immediately before the start of the pilot (Time 1); immediately post-intervention (Time 2); and at a 2 month follow-up (Time 3). The qualitative part of Study 2 consisted of semi-structured interviews about participants’ experience of the pilot training (*n* = 21) at 2-month follow-up.

### TMT Intervention Design

The core aim in designing this pilot “next generation” MBI is to maximize the potential of mindfulness to help individuals and teams manage work stress better, both individually and collectively.

More specifically, TMT integrates the five key processes of collective mindfulness (Weick et al., 2000) with the five key components of individual-level mindfulness practice deemed essential to form a genuine (individually focused) MBI: contemplative mindfulness practices such as observing the breath; a model of human experience explaining the causes of distress and how to relieve it; facilitating an approach-oriented and de-fused relationship with experience; promoting self-regulation of attention and positive qualities such as wisdom and compassion; and engaging participants in sustained mindfulness meditation practice and inquiry-based learning to develop insight (Crane et al., 2017).

#### Structure of TMT

The structure of the TMT mirrored MBSR for ease of comparison in the pilot, with individuals participating for 2 h in weekly sessions over an 8-week period. This choice reflected the need to keep the design of TMT comparable to MBSR (rather than a careful examination of the theoretical constructs under study before designing the intervention; this was not feasible because of the need for a comparative pilot evaluation).

In each training session, roughly half of the time was devoted to developing individual mindfulness-based stress management skills, as taught in the well-researched MBSR curriculum (Blacker et al., 2009). Participants were invited to practice both formal mindfulness meditation practices as well as to engage in individually focused reflections on how to manage personal stress more effectively, integrated into their lives and in ways that fit their specific context. As per prior research in the United States Military (Jha et al., 2015), this aspect of the intervention was only very minimally adapted from the MBSR protocol by Blacker et al. (2009).

The individual-level mindfulness training proportion was cut in half because of emerging evidence that shorter versions of the MBSR curriculum may be as effective as the originally developed version (Creswell, 2017). This may be particularly appropriate for work teams (Carmody et al., 2009), and enables a delivery team to keep the total contact time in line with standard expectations for “traditional” mindfulness training.

As Crane et al. emphasize in their (2017) article stipulating the five essential components of MBIs, adaptations based on relevant theoretical frameworks and tailored to a particular population (such as individuals working and serving in the military, as in the present context) are encouraged. This served as platform for the creation of the collectively focused part of TMT.

Therefore, the remaining half of training time consisted of collective mindfulness training elements (this time split was chosen predominately because it meant that TMT fit within the standard timeframe of MBSR).

Specifically, TMT participants were invited during each session to engage in experiential exercises and small group discussions, focused on developing collective stress management capacity in line with the five collective mindfulness hallmarks.

However, scholars agree that collective mindfulness emerges *indirectly* as a consequence of a particular “heedful” (Weick and Roberts, 1993) and prosocial way of interacting (Vogus et al., 2014). Interventions targeting a particular collective mindfulness hallmark *directly* are unlikely to be effective because collective mindfulness depends on leadership and culture that reflects social agreement on valuing mindful collective practices. By way of example, imagine you belong to a team, with an existing hierarchy of team leader and followers, based on rank and career history. Inviting you to defer decision power for each future team decision to the most knowledgeable individual independent of their position in the hierarchy would involve revisiting the team’s established leadership and culture *before* this collective mindfulness hallmark can emerge.

Therefore, the five collective mindfulness hallmarks were used as indirect target outcomes for weekly interventions that directly promoted prosociality and open-mindedness at an interpersonal level, based on the assertion of Vogus et al. (2014) that these serve as affective foundation of collective mindfulness. At the interface between the individual and their relationship with others, this feature of the training is based on the observational insights of Fraher et al.’s (2017) study of United States Navy SEALs, suggesting that trust-based, pro-social and respectful work relationships foster multi-level “mindfulness in action.”

#### TMT Session Content

Each session of the TMT focused on a specific topic.

The first session included a general overview of scientific evidence linking mindfulness with resilience and sustained performance at individual and collective levels in organizations. The starting point for the collective part of the TMT program was illustrating why and how mindful organizing, that is anticipating and responding to unexpected stressful challenges collectively, rather than individually, may benefit individual participants in their specific setting by helping them improve individual resilience and performance as well as the overall reliability of the entire team.

The session linked mindfulness with psychological safety, a shared belief that the team is safe for expressing suggestions or ideas that are important to the individual yet that might make him or her vulnerable to being ostracized from the group, and one of the most reliable predictors of team performance (Edmondson, 1999). Psychological safety overlaps significantly with team mindfulness (Yu and Zellmer-Bruhn, 2018). In the session, participants were inductively led to reflect on their own preconceptions about the link between psychological safety and team performance, exploring how this research looks and sounds in their own context. Subsequently, they were invited to discuss in small teams how they may collectively overcome the emotional difficulty involved in considering, let alone proactively discussing in a work team setting, fears of failure during a concrete upcoming stressful challenge. The overall intent of this collective mindfulness exercise was to generate motivation and commitment to responding to upcoming stressors as a collective unit, helping each other improve resilient performance under stress and pressure both individually and collectively.

Session 2 introduced the idea of situational as well as self-awareness, when tackling work problems and challenges. This session centered around the collective mindfulness hallmark “sensitivity to operations” brought to life through inductive exercises intended to gently help participants question their own assumptions about the types of information they tend to communicate (verbally and non-verbally) and the information they tend to focus on when interacting with others, especially when under stress at work.

Session 3 focuses on the collective hallmark “preoccupation with failure,” continuing to jointly uncover the automatic judgments people at work tend to make about important normal aspects of work life, such as failing to succeed 100% of the time. Participants were invited to consider both positive and negative aspects that the topic “failure” brings up for them individually and in group discussions, in order to prompt a shift in perspective and a changed relationship with this topic in their own work lives towards embracing difficulty at work in a more accepting, proactive way. Groups were invited to continue shifting their focus of attention in relation to this topic by exploring positive aspects that go hand in hand with perceptions of “failing” in their world of work, such as learning.

Session 4 helped participants develop a greater collective appreciation for “reluctance to simplify,” in other words changing their relationship with complex work challenges. Participants were invited to bring this idea to life by engaging in a traditional group mindfulness exercise, based on the teachings of Levey and Levey (2014), sharing personal values and exploring where these overlap or diverge, and what this meant for individuals and the group as a whole. Leaning on Ely and Meyerson (2010), this exercise is linked to creating team micro-cultures of other orientation, by inviting the participants to explore how to create team cultures in which everyone serves as their proverbial brother’s keeper to succeed, rather than reinforcing a work culture of proself motivation.

In session 5, the collective mindfulness theme was “deference to expertise,” and here participants were invited to reflect in small groups on the positive and negative aspects of being considered an expert, and comparing and contrasting real-time rather than historically determined expertise. The aim in this was to open participants’ minds towards looking for expertise in their work teams that may not align with traditional expectations of expertise (based on age, gender, rank, and so on). In this context, they were also invited to map out their personal social network, graphically illustrating who they communicate with for different needs, and encouraged to strengthen their social network further with a view to uncovering innovative sources of social connection and expertise.

Session 6 was focused on the collective mindfulness hallmark “commitment to resilience,” encouraging adaptability in the face of unexpected challenge. At the heart of the inductive learning for this session was an exploration of the drivers and effects of unexpected interpersonal conflict, brought to life through role plays and group reflections. Based on Grant and Berry (2011), who found that prosocial motivation leads to individuals considering others’ perspectives and questioning their own assumptions, this session focused on perspective-taking exercises (taken from Flaxman et al., 2013), to help participants strengthen their interpersonal awareness and emotional intelligence.

Session 7 brought together all collective mindfulness skills learning through a group exercise inviting participants to engage in a Pre-Mortem (Klein, 2007) about a significant future challenge specific to their own work context, thus playfully changing perspective and collaboratively planning how to avoid being unsuccessful in facing this real-world challenge.

The final session prepared participants for embedding mindfulness into their personal as well as their teamwork realities, leaning on standard MBSR curriculum recommendations for completing the MBI learning cycle.

Sessions were arranged so that participants worked in small groups both during the sessions as well as in self-organized fashion between sessions. Participants were invited to continue practicing what they have learned between sessions, by engaging in short pre-recorded daily mindfulness meditation practices as well as through 1–2 team exercises aimed at embedding collective mindfulness routines. In total, daily mindfulness “homework” meditations were kept to 10–15 min, to not overburden participants in their time commitments to practicing mindfulness between sessions, as they were also encouraged to meet with their fellow participants for 1–2 h between each session.

#### Delivery Approach

Because MBIs touch upon exploring human suffering, the TMT was delivered by two facilitators, one of whom is a trained therapist. This is in line with general practice in MBSR and related mindfulness programs (e.g. Neff and Germer, 2013), to be prepared for situations in which a participant may require the attention of a psychotherapeutically qualified facilitator.

In the present study, three experienced mindfulness facilitators alternated co-facilitating the training. Two of these had a long-term personal practice of mindfulness meditation (more than 10 years each) and extensive experience of teaching mindfulness to high-performance work populations. The third facilitator was a collective mindfulness expert and former military officer with extensive experience of providing military training.

### Research Setting

The study was sponsored by the United Kingdom Ministry of Defence (MoD). The duration of the study was 21 months. The MoD appointed a steering group made up of military and civilian stakeholders with interest in the study; the military stakeholders represented all three Services of the United Kingdom military. The research team, headed up by the first author, consisted of three mindfulness trainers and three evaluation experts, and met with the steering committee on a quarterly basis to present and discuss the study’s progress and findings.

During the first 6 months of the study, the researcher team conducted a series of site visits, informal interviews, presentations, and workshops at all three branches of the United Kingdom military, to ascertain the study context, to obtain initial feedback on the TMT design, and to determine two suitable and comparable host sites for the two planned research studies. Two sites were selected for Study 1 and 2, respectively: A British Army initial military officer training site (for Study 1), and a Royal Navy initial military officer training site (for Study 2). Both sites offer equivalent military training at two different Services of the United Kingdom Armed Forces.

These two sites were chosen because initial military officer training in the United Kingdom in each Service takes place mostly in the same location; is designed to be physically and mentally challenging over a sustained period, with notoriously little time to relax; and it simulates a diverse range of intellectual and professional performance trials for future leaders of an organization facing increasingly complex challenges in the twenty-first century.

Furthermore, a military setting was apt for a trial of “next-generation” mindfulness because its strong culture of self-sacrifice and high dedication stood in potential conflict with a perception of mindfulness as self-help (Choi et al., 2020), thus approaching mindfulness training *as team sport* was considered potentially more fit for purpose.

Arguably, there are some parallels that can be drawn between the military context and the high-stress environment that numerous workers in today’s conventional public and private sector organizations find themselves in at one time or other.

The research was subject to review by the Ministry of Defence Research Ethics Committee (MODREC), who gave a favorable opinion. Ethical approval to conduct the studies was also obtained from the first author’s university ethics committee.

### Data Analysis Strategy

The first author led the intervention and data analysis (but not the data collection; this task was completed by the evaluation team members). To ensure trustworthiness of the data analysis, the first author referred to a reflective journal they had built throughout the study, reflecting on the relationships, interactions, contextual understanding, and potential biases that may have formed during this experience, and recognizing that the researcher brought into the analysis their personal understanding of the study’s context, built over numerous prior site visits, meetings, and informal discussions (Boyatzis, 1998).

A deductive thematic data analysis approach (Braun and Clarke, 2006) was applied to both sets of qualitative data, to focus the data analysis effort specifically on answering the research question in terms of improving participants’ individual and collective stress management skills.

Study 2 was also evaluated quantitatively using regression analyses including effect size calculations, to answer the study’s RQ whether and how TMT can generate individual and collective stress management skills, and compare its effectiveness against MBSR, serving as an active control condition.

## Study 1: Pre-pilot

### Method

Study 1 was a pre-pilot intervention of TMT with British Army officer cadets in initial training (*n* = 23). The pre-pilot was evaluated qualitatively using a pen and paper survey (*n* = 21; two training participants declined to join the data collection effort) immediately upon completing the pre-pilot training intervention.

### Participants and Procedure

All potential participants were at the start of their military career. Invited participants had been extensively pre-screened for mental health concerns by the host establishment prior to commencing initially military training; hence, standard MBI pre-screening was omitted (individuals with known mental health conditions had been excluded from military training). Eligible individuals were at least 18 years old and had been briefed about study participation at least 5 days before taking part.

Participants were drawn from one particular division at a British Army officer training establishment (all volunteered to participate in the training). There were 18 males and five females, 100% Caucasian, with a mean age of approximately 23 years. Timetabling constraints meant that the training occurred over 10 weeks, not 8 as intended.

### Measures

Pre-pilot survey questions were administered at the end of the pre-pilot. They were open-ended and invited participants to reflect in writing about their overall impression of the program; what was positive; what was negative; how the program could be improved; and any other comments they would be prepared to share.

### Results

Table 1 shows a summary of the three thematic codes used for the data analysis, alongside subthemes and illustrative quotes from interviewees. “Suggestions” served as third semantic backbone for the organization of findings, as this summarized additional relevant feedback from participants. Quotes are attributed to interviewed participant by adding a randomly allocated identifier 1, 2, etc., to each participant, which means that (P1) refers to Participant 1, (P2) to Participant 2, and so on. Each theme is illustrated further below.

**Table 1:** Study 1 qualitative themes and subthemes alongside illustrative quotes.

Thematic code	Subtheme	Illustrative quotes
Individual stress management	Self-awareness	“Recognizing emotional discomfort is the first step to managing it.” (P13)
	Individual stress reduction skills	“When I have felt frustrated or annoyed after something has occurred, I have used some of the mindfulness exercises to control my emotions, be more composed and think more clearly.” (P16)
	Attitude change	“I can conquer things I never thought possible, mainly myself.” (P6)
Collective stress management	Social awareness	“Everyone has more or less the same fears/doubts about failure as I do, and is equally invested in success.” (P10)
	Openness towards difference	“You have to give everyone a chance to contribute, everyone has different styles and ways of seeing things that can be invaluable.” (P18)
	Helping others feel safe	“The most essential ingredient of effective teamwork is that everyone in the group feels comfortable.” (P15)
	Collective stress management skills	“It’s better to manage it than to leave things unsaid!” (P2)
Suggestions	Less individual contemplation	“Less on individual coping techniques – much of it is easily accessible through apps like Headspace.” (P12)
	More team mindfulness	“Putting more emphasis on the team mindfulness early on, so that we might practice it more.” (P4)
	Focus on participants’ context	“Understand our situation and tailor it for us.” (P2)

#### Individual Stress Management

Three sub-themes emerged in this context: (a) self-awareness; (b) individual stress reduction skills; and (c) attitude change.

First, virtually all individuals in the sample suggested that their conscious awareness of themselves and their emotions had increased. In this context, an increased mind–body awareness was frequently coupled with a sense of helpful experiential acceptance, such as the following

“*Awareness of my body and why it reacts. Realizing it’s normal not weak.*” (P7).

In addition, the majority of training participants seemed to have learned skills to cope with stressful moments. Sixteen cadets mentioned “*better stress response techniques*” (P9) and methods “*to deal better with my worries and anxiety*” (P11). Insights ranged from “*skills taught to avoid stress*” (P6) to learning how to handle stressful situations more effectively. Furthermore, a sense of optimism seemed to imbue several reflections, such as “*I do not just have to cope with change, I can thrive in it*” (P4).

#### Collective Stress Management

The following four subthemes emerged on the impact of the TMT on participants in relation to others in the team as a whole: (a) social awareness; (b) openness towards difference; (c) helping others feel safe; and (d) collective stress management skills.

The first subtheme revolved around heightened social awareness, centered around *being* “*more mindful towards others*” (P5) and “*understanding others’ feelings*” (P1). Several cadets indicated they had learned to be aware of body language and “*noticing non-verbal contributions*” (P12). In particular, several cadets expressed a more conscious perception of how essentially similar their thoughts and feelings were to those of their fellow team members. The quote below illustrates this:

“*Whilst everyone in the team is very different and brings different elements, we all have similar thoughts/goals.*” (P16).

Next, there seemed to be a recognition that it might be helpful to “*be more aware of everyone around me, not just those I naturally gravitate towards*” (P7). Twelve individuals discussed how the training had prompted openness towards difference. This openness expressed itself as an increased awareness of “*emotional discrepancies*” (P14), as well as an acceptance of different perspectives: eight cadets reflected on how the course has made them “*more aware of the way other people think*” (P9), and that “*people expect or want different things*” (P10).

Moreover, about half of the participants shared insights they had learned on effective teamwork. Six individuals indicated they had learned to become “*a better team leader and follower*” (P12) and spoke of a heightened awareness of “*how to help others and how to maximize team potential*” (P11). Specific examples of this focused on reflections about drivers of team effectiveness, outlining their “*understanding what the key attributes are a team truly needs*” (P16). This seemed to have impacted prior expectations of effective teamwork, as the statements below suggest:

“*Intuitively you expect factors like structure and clarity to be the crucial ones when actually verbal turn-taking and discussing body language can be more important.*” (P8).

Finally, several officer cadets noted a change in understanding of how to draw on others to help manage stress. In particular, four individuals mentioned they had learned “*to be open and share vulnerability*” (P17). One officer cadet spoke of an “*acceptance about opening up to a group and teammates and how it can help me*” (P7), suggesting that this openness was deemed beneficial.

#### Suggestions

Participants’ suggestions for improving TMT were grouped into three sub-themes: (a) less individual contemplation; (b) more team mindfulness; and (c) focus on the participants’ context.

First, participants’ feedback seemed to indicate a slight preference for socially focused, action-oriented learning. One officer cadet noted they preferred such “*more practical parts*” (P15). This preference may also be linked to the following perception:

“*Much of [individual mindfulness] is easily accessible through apps like Headspace.*” (P12).

Additionally, some participants requested “*more focus on teamwork and social dynamics*” (P8). Two officer cadets indicated they found this aspect of the training particularly useful:

“*Team mindfulness was something I struggled to understand initially, and I wish it had been more central to the course earlier on.*” (P15).

Finally, about a third of all participants suggested a more pronounced emphasis on tailoring the course to their particular work context, in short; “*understand our situation and tailor it for us*” (P2).

### Summary

Results from the pre-pilot study evaluation were encouraging. Above all, survey participants reported generally positive perceptions about the training, relating a range of new insights and learning they linked back to the professional challenges in their work context. In addition, participants seemed to welcome the addition of the newly designed TMT components of the program, to help them manage stress better, not only individually, but also collectively. Finally, improvement suggestions revolved largely around contextualizing the training content more and changing the ratio of individual as opposed to team mindfulness practices. Some individuals even seemed to express a slight preference for team-based mindfulness practices over “classic” individual mindfulness meditation practice. Some caution should be exercised, however, given that fewer than a handful of individuals explicitly compared individual with team mindfulness in their feedback.

These data provided preliminary support for the argument that the TMT program may show potential. While these results were promising, the study’s major limitation was twofold: First, no quantitative outcomes had been collected in Study 1, to ascertain any changes in stress management capacity linked to participating in the training. And second, the study had not been set in context with a control group. To investigate the potential of the TMT program further, Study 2 was conducted.

## Study 2: Mixed Method Controlled Pilot

### Method

Study 2 was a second trial of the TMT program: a formal mixed-method pilot study comparing TMT to a minimally adapted version of MBSR serving as an active control condition. The study implemented a 2 (TMT vs. MBSR) × 3 (baseline; post; follow-up) block randomized study design for the quantitative part, yielding a between-groups comparison of the TMT program (*n*=105).

The qualitative part of this study consisted of semi-structured interviews conducted over the telephone at the same time as collecting the quantitative follow-up data, inviting participants to share their experience of the program (*n*=21; two of these withdrew their consent to have their data analyzed subsequently). Both parts contributed to answering the study’s RQ in comparison with “classic” mindfulness training based on MBSR.

### Participants and Procedure

Royal Navy officer cadets in training at the beginning of their military career were invited to participate. Sampling procedure was identical to Study 1. Eleven individuals from the sampled population chose not to participate in the training intervention. Participants had been clustered into four military training divisions. Each division worked, trained, socialized, and prepared for performance assessments together; hence, a division served as proxy for a work team. Participants remained blind to the two conditions throughout Study 2. Both intervention types were named “mental fitness training” although it was made clear that all training was based on mindfulness science. Cross-group contamination was unlikely as divisions operated in friendly competition with each other. Each division had a similar composition of participant demographics, in that the training establishment had purposefully counterbalanced the divisions along demographic characteristics such as number of university graduates, females, age, years of work experience, individuals whose first language is other than English, and so on. The average age was approximately 23 years. There were 15 female participants.

The intervention group participated in the TMT program described earlier, after minor changes had been made to language and content, as per Study 1 participants’ recommendations. The control group participated in a slightly adapted version of MBSR (notably omitting the mindfulness retreat, shortening the session length, and situating exercises in a military work context). Both conditions were led by the same instructors who had also led the pre-pilot sessions in Study 1. Training occurred weekly (with a 2-week gap between Session 4 and 5, because of operational constraints at the host site) over a 10-week period, conducted sequentially for the two intervention types, but alternating which of the two groups would be trained first each week. Session length was kept identical for both conditions.

### Measures

#### Quantitative Measures

The following three validated standard measurement scales were included (it was not acceptable for the host institution to request more time commitment for study testing from participants).

##### Individual Resilience

Individual resilience was assessed using Campbell-Sills and Stein (2007) 10-item CD-RISC scale which has reasonably good psychometric properties. This scale was used because it is a shortened version of the most widely used assessment of resilience worldwide (Rees et al., 2015).

##### Mindful Organizing

Participants’ perceptions about MO in their division were captured using the nine-item Safety Organizing Scale, assessing Weick et al.’s five processes of collective mindfulness (Weick et al., 2000; Vogus and Sutcliffe, 2007b). The scale has high internal reliability (*α* = 0.88; *ibid.*), as well as high discriminant validity in relation to related concepts such as organizational trust and commitment (Ausserhofer et al., 2013). Responses are given on a 7-point scale ranging from 1 (*not at all*) to 7 (*to a very great extent*). High scores on the Safety Organizing scale are linked to low organizational errors; for example, a one-point increase in Safety Organizing corresponds to almost 30% fewer hospital medication errors 6 months later (Vogus and Sutcliffe, 2007a). Total scores for Safety Organizing range from a minimum of 9 to a maximum of 63. Total scores are calculated by summing scores on all nine items. A higher overall score indicates higher self-reported MO. Examples of statements include “*We spend time identifying activities we do not want to go wrong*” and “*We talk about mistakes and ways to learn from them*.”

##### Working Memory

Individual performance under pressure was operationalized using the working memory (WM) test by Unsworth et al. (2005). This is an objective computer-based test, measuring an individual’s WM over a series of timed trials during which the participant needs to solve simple math problems while remembering and recalling letter sequences. WM is used in managing cognitive demands and regulating emotions, and WM span tasks have been shown to predict performance during demanding cognitive challenges (e.g., Engle et al., 1999; Kane et al., 2001). WM is a key component involved in cultivating cognitive mindfulness processes (Jha et al., 2010). Prior mindfulness research in military settings suggested that those with low rather than high WM capacity are more likely to suffer from emotionally intrusive thoughts, and used the same type of measurement to assess individuals’ performance (Jha et al., 2010, 2015). Unsworth et al. (2005) showed that their WM measure has both good internal consistency (*α* = 0.78) and test–retest reliability (0.83), and they report that it correlates with other measures of WM capacity (construct validity). The top score possible is 75 and general population samples achieved scores ranging from 28 in the lower quartile to 66 in the upper quartile (*ibid.*).

#### Qualitative Measures

Participants were invited to share their perceptions of the program using semi-structured interviews with members of the data collection team, including what was positive; what was negative; how the program could be improved; and any other comments they would be prepared to share.

### Results

#### Quantitative Results

Statistical analyses were conducted to examine the comparative effectiveness of the two intervention types by determining the effect of each intervention type, using SPSS version 25 (IBM Corp, 2017). The grouping variable represented the different intervention groups and had two levels (“Individual” vs. “Team” mindfulness training), and the within-group variable was “time” with three levels (Baseline [T1], post-intervention [T2], and 2-month follow-up [T3]).

Descriptive statistics are provided in Supplementary Material. First, it was examined whether there were significant differences between the intervention and control group on any demographics or measures at pre-test, and none were found.

The analyses further investigated whether there were any differences in outcomes between the two training types by comparing outcomes using a series of 2 (Group) × 3 (Time) repeated measures analyses of variance.

##### Individual Resilience

A significant main effect of “time” for individual resilience for both groups (*F*[1.713, 122] = 8.666, *p* = 0.001) was found, with a large effect size (*n*_*p*_^2^ = 0.124; Cohen, 1988) immediately after the groups had completed the interventions. In other words, a significant improvement in self-reported resilience could be detected. Participants in both groups reported significant increases in resilience immediately after the intervention had been completed (*F*[1, 61] = 16.576, *p* < 0.001, *n*_*p*_^2^ = 0.214). There was no significant difference between the groups (*F*[1.713,122] = 1.733, *p* = 0.78, *n*_*p*_^2^ = 0.004). This improvement was maintained 2 months after the intervention had been completed (*F*[1, 61] = 5.109, *p* = 0.027, *n*_*p*_^2^ = 0.077; medium effect size, Cohen, 1988). However, there was no significant difference between T2 (post-intervention) and T3 (2-month follow-up; *F*[1, 61] = 3.794, *p* = 0.066). In terms of magnitude of effect size, this may be a medium effect.

The data appear to show a ceiling effect of the self-reported resilience scores at baseline.

##### Working Memory

For working memory, there was a significant main effect over time, (*F*(2, 120) = 3.958, *p* = 0.022), with a medium effect size (*n*_*p*_^2^ = 0.062; Cohen, 1988). As above, there was no significant different between groups, (*F*(2, 120) = 0.534, *p* = 0.588, *n*_*p*_^2^ = 0.009). Participants in both groups reported significantly better working memory immediately after their interventions had been completed, (*F*(1, 60) = 5.747, *p* = 0.02, *n*_*p*_^2^ = 0.087), but these gains were not maintained at 2-month follow-up with both groups reverting to levels similar to those reported at baseline (*F*[1, 60] = 6.967, *p* = 0.011, *n*_*p*_^2^ = 0.104).

This data trend also seems to suggest that the 8-week mindfulness training format may be insufficient for meaningful cognitive gains in operational contexts to sustain.

Taken together, these analyses indicate that the TMT program appears as effective as the “standard” individual focused MBSR program in generating beneficial change in individual resilience and cognitive performance under pressure.

##### Mindful Organizing

**Figure 1:**
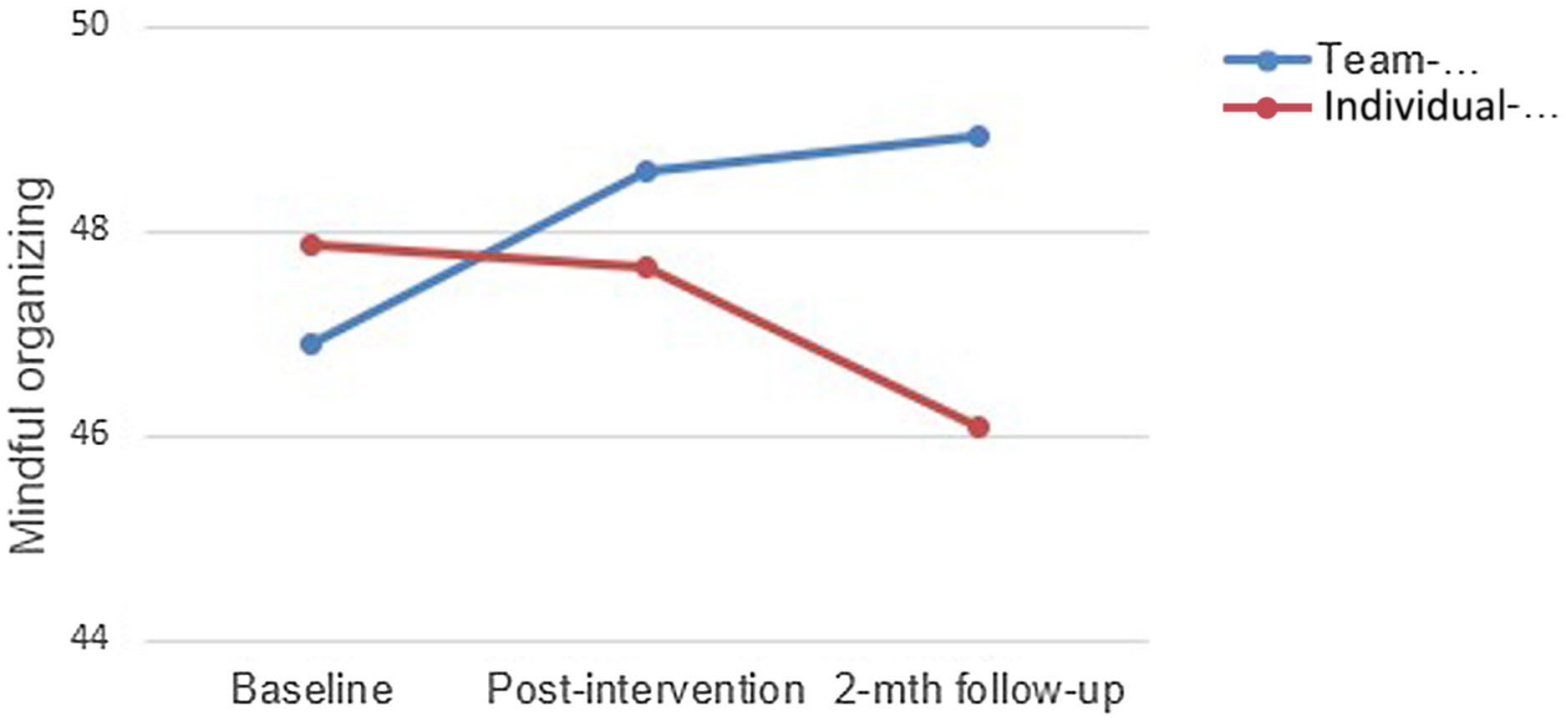
Measures of mindful organizing over time for “Team” and “Individual” groups.

There were no significant effects in the data for MO. However, there was a trend in the data suggesting that the two groups were differentially affected by their respective interventions, with the “Team” group demonstrating increased levels of MO over time, a trend that was not evident for the “Individual” group (see Figure 1).

Despite the absence of significant difference, the data trend for the intervention group seems to indicate that TMT may be more beneficial than “classic” MBSR in generating collective stress management skills. Theoretically, one would expect team-level perceptions of change to take longer to develop than over the course of several weeks; it takes time for teams to work together effectively in any work context. We will return to this point in the Discussion.

#### Qualitative Results

The same qualitative research method as per Study 1 was applied to help answer the RQ at the heart of this inquiry. Overall, participants from the TMT condition seemed to be able to discuss learnings that related to generating collective stress management capacity, more so than participants in the MBSR condition. This was in line with the rationale for conducting the present study in the first place. However, it is noteworthy that only individuals from this group indicated that they were able to apply their newly learned individual stress management skills during a highly stressful performance challenge during their officer training, i.e., when they most needed stress management skills. This suggests that the collective elements of the training might have created a collectively mindful team climate that welcomed the open application of such skills.

In Table 2, summarizing thematic codes, subthemes, and illustrative quotes or notes, quotes are attributed to individuals by their respective training condition using the following notation: I1 would refer to participant 1 in the Individual condition, while T2 would refer to participant 2 in the Team condition, for example.

**Table 2:** Study 2 qualitative themes and subthemes alongside illustrative quotes.

Thematic code	Subtheme	Illustrative quotes
Individual stress management	Individual stress reduction skills	“learning to be calm in stressful situations” (T12)
	Attitude change	“I have learnt not to worry about [critical] comments because I cannot control what other people say but I can control how I react to them” (T6)
Collective stress management	Social awareness	“The training made us think more about how we communicated with each other” (T2)
	Practicing mindfulness together	“‘Right, everyone takes a deep breath’, and sometimes four or five people will do it together for a few seconds.” (T9)
	Collective stress management skills	“[we] would no longer be talking over each other during tasks” (T2)
Suggestions	Focus on participants’ context	“need to have someone from the Forces to ‘sell’ the course” (I5)
	More team mindfulness	“The team building element was the main benefit out of all this” (T7)

##### Individual Stress Management

Interview data indicated that participants found the training valuable for managing their personal stress better, relating back work occasions where they had applied what they had learned to good effect. Many related that the mindfulness training helped them learn “*to be calm in stressful situations*” (T12), especially when their performance was tested, for example, giving presentations in front of senior audiences, before which many individuals would become nervous. One interviewee explained that a number of their team members would use the breathing techniques to calm themselves down, adding “*you could see that even during the presentation they were doing the breathing*” (T12). Several interviewees mentioned that noticing their response to stressful challenges had helped them change their attitude towards such stressors, recounting in particular that they had found it beneficial to learn how to label their emotions in order to let go, and get on with the task at hand. One officer cadet summed this up as follows:

“*[Even] if you cannot always change the way you feel at the time, you can always notice how you react to things*” (T1).

##### Collective Stress Management

Half of the interviewees from the TMT group identified team-focused benefits, including (a) social awareness; (b) practicing mindfulness together; and (c) collective stress management skills.

First, several officer cadets indicated that the training helped increase their social awareness of others and their needs, especially in preparation of stressful challenges ahead. One interviewee suggested that the training prompted them to do the following:

“*The training made us think more about how we communicated with each other*” (T2).

Second, it is noteworthy that only participants in the TMT condition shared experiences of collectively practicing individual mindfulness techniques with others, to help calm themselves down. For example, one cadet mentioned using the mindful body scan technique to help them relax with their roommate, explaining they “*both would be doing that in the evenings*” (T3). Other situations were mentioned during which one course participant would suggest practicing mindfulness together, including routine performance inspections during their day-to-day work. An interviewee recalled that sometimes a person would remind the group as they were lined up and waiting for the inspection to begin:

“*‘Right, everyone takes a deep breath’, and sometimes four or five people will do it together for a few seconds.*” (T9).

Finally, about a handful of interviewees reflected on learning from the training in the context of a major performance challenge that everyone had to complete shortly after the end of the training intervention, assessing individual and team performance over a period of several days and nights outdoors. Virtually every one of these individuals spoke of how much stress and conflict this performance challenge provoked in their teams on a continuous basis. For example, one interviewee recounted a situation during the performance challenge when the team was lost, which meant everyone in the group felt stressed. One of the team members spoke up and identified that team members were becoming argumentative and rash with each other, so the team “*decided to collectively do some breathing exercises together*” (T10). This, the interviewee suggested, helped the team members calm down and work out how to resolve the situation. Yet another interviewee related a conflict the team faced during the performance challenge, during which they used one of the team mindfulness techniques from the training to give everyone a say, openly and constructively discussing the problem. In the words of the participant:

“*It really helped the group perform better – [we] would no longer be talking over each other during tasks.*” (T2).

While these comments indicate that TMT participants clearly experienced collective benefits, only five individuals specifically highlighted the benefits of learning to develop collective stress management capacity. Several of these suggested a focus on team development and communication “*definitely made a difference*” (T2).

In stark contrast to this, none of the interviewees from the MBSR group were able to recall any collective benefits of the mindfulness training to the division as a whole. One participant said:

“*We were not much of a team so would not really expect to see team benefits.*” (I3).

##### Suggestions

Two subthemes are discussed here: (a) focus on participants’ context; and (b) more team mindfulness. First, besides a variety of recommendations concerning session length (some suggested shorter, more frequent, training sessions, while others would have wanted longer sessions and train for a longer time), there was a general sense that the training should be more focused on the participants’ context. The training set-up should “*clearly show the relevance of the training*” (I3) to the participants’ specific work situation, and future training organizers would “*need to have someone from the Forces to ‘sell’ the course*” (I5).

Second, and in parallel to Study 1, there was an indication that team-focused mindfulness aspects of the training was welcome and should have been a more central feature of the training. The quote below sums up this sentiment:

“*The team building element was the main benefit out of all this. Although perhaps we could have got to that aspect a bit quicker. I would have liked even more of it. It means that as a division we were ready for what was thrown at us. We did not have lots of hassle, we could trust in each other, take a collective deep breath, and get on.*” (T7).

### Summary

Results from Study 2 extended the insights from Study 1 on TMT’s potential and point to the following key findings: First, it appears that TMT seems no less effective in raising individual stress management skills than a “first-generation” MBI. In addition, the qualitative investigation of participant experience across both studies suggests that TMT may hold more promise in generating collective capacity to manage stress and difficulty.

Most intriguing, however, is the second key finding; an apparent interdependence between individual and collective mindfulness capability, specifically that collective mindfulness training element seemed to facilitate individual mindfulness capacity development. In the present study, an emerging collectively mindful team climate in the TMT group seemed to enable the application of individual mindfulness skills during moments of high stress and challenge. This means that when mindfulness was embedded in team relationships and social interactions, it seemed easier for individuals to apply newly learned mindfulness-based stress management techniques, especially when these were particularly needed. In contrast, the officer cadets in the “first-generation” MBSR training condition struggled to recall any benefits other than using techniques to fall asleep, and none mentioned using the techniques collectively.

A discussion of how these findings contribute to the theory and practice of “next-generation” mindfulness training for organizations is provided below, starting with the second key finding first.

## Discussion

This inquiry opened with Kabat-Zinn’s humble assertion that MBSR is but one of a potentially infinite “skillful means” to bring wisdom into the world (Kabat-Zinn, 2011). The research question guiding the inquiry was whether TMT can generate individual and collective stress management skills, and if so, how.

The study’s research approach consisted of combining two previously siloed mindfulness literatures in order to investigate untapped synergies and to stimulate debate on appropriate new ways to “blend” them (Oswick et al., 2011), rather than continuing to adapt “first-generation” MBIs.

This reflects a paradigm shift in the science of evidence-based interventions from named therapies (e.g., MBSR) to process-based approaches (Hofmann and Hayes, 2019). Process-based intervention science focuses on asking questions such as “what processes should be targeted, for whom, how, and in which context?” in order to design evidence-based processes and contextually specific means to improve outcomes for people (*ibid.*).

Based on the conclusions from the empirical work this study makes the following contributions to theory-building on the transformative potential of mindfulness training particularly in workplaces: (1) Individual and collective mindfulness in workplaces may be interdependent; and (2) mindfulness training beyond meditation may extend its transformative potential.

Interdependence theory is used to structure this discussion. Interdependence theory examines the role of social orientations (e.g., cooperation or conflict) in situations of interpersonal outcome interdependence (Kelley and Thibaut, 1978). This structure was chosen for two reasons.

First, while a detailed understanding of intrapersonal (or intrapsychic) processes is important to advance mindfulness science, interpersonal processes and their impact on people’s reality, motivation and behavior should not be ignored (Rusbult and van Lange, 2008), especially in work contexts. In other words, these processes create the emotional context in which individuals navigate their choices and action.

And second, interdependence theory can help create new linkages between mindfulness theorizing and the strong focus on ethical and contextualized conceptualizations of mindfulness (c.f. Purser and Milillo, 2015) and on other-orientation and interdependence in contemplative tradition that may have been overlooked in contemporary debates on mindfulness (c.f. Gergen, 2009). As the Dalai Lama argues, great wisdom emerges when people become “able to appreciate the interdependent nature of one’s own and others’ interests” (Dalai Lama, 2005, p. 109).

### Interdependence Between Individual and Collective Mindfulness in Workplaces

With specific focus on work settings, the empirical results of this study point to an important yet understudied phenomenon in mindfulness research: when comparing a standard “first-generation” mindfulness intervention (targeting predominately individual mindfulness-based stress reduction skills) to an intervention that included a focus on collectively mindful relationships and interactions among participants, it seems that “first-generation” mindfulness training alone may be less effective in generating the “living foundation” (Kabat-Zinn, 2011, p. 7) that enables individuals to develop an embodied mindfulness practice *in situ* at work and draw on mindfulness especially when facing stress.

These findings contradict the long-held assertion that a “living foundation” for mindfulness can only be cultivated *from the inside out* (Kabat-Zinn, 2011). It would appear that in a work context, the transformative potential of mindfulness to generate wisdom and compassion for the self and for others may also be cultivated *from the outside in*, by promoting collective mindfulness, which in turn may promote individual mindfulness.

There are at least three reasons for observing this phenomenon.

First, collective mindfulness enables individuals to interact more “heedfully” (Weick and Roberts, 1993). This means they are able to: (a) balance their attention between self- and situational awareness; (b) shape and adjust individual action in line with the needs of others and the overall situation; and (c) create and maintain strong connections and learn from each other (*ibid.*). Therefore, work teams that organize mindfully have a mindful culture (Sutcliffe et al., 2016). The affective foundation for such a collectively mindful culture includes prosociality (Vogus et al., 2014), synonymous to the practice of compassion in the Buddhist tradition. Based on this foundation, a work culture can emerge that welcomes care and concern for individuals’ wellbeing as much as focusing on performance outcomes. We thus speculate that collective mindfulness skills development may therefore be an important enabler of individual mindfulness, especially in work contexts where self-care may be deemed counter-cultural or frowned upon. Conversely, in the absence of a collectively mindful culture, individual mindfulness is less likely to thrive.

Second, interdependence and in particular prosociality not only benefits others but also improves the actor’s own wellbeing (Aknin et al., 2013; Klein, 2017). Social engagement is a powerful antidote to stress, both by reaching out to others for help when stressed, and also by providing empathy and comfort to those feeling stressed (Porges, 2011). In other words, (work) stress may validly be reduced from the outside in as well as from the inside out. Therefore, training programmes such as TMT that promote social engagement as part of developing a mindful culture can extend the potential of mindfulness to transform how workplaces manage stress, individually and collectively.

Third, collective mindfulness generates metacognitive capacity (Kudesia, 2019)—at a collective level—because it is focused on *assumption management*. In other words, collective mindfulness helps shift a team’s information processing style to deliberate engagement in the service of proactively identifying and examining the underlying assumptions that may prevent or facilitate resilient performance under stress and during unexpected challenges (Weick and Sutcliffe, 2007). A collective increase in metacognitive capacity can therefore facilitate—and legitimize—improved metacognition within individuals, a particularly valuable mindfulness skill for people at work (Kudesia, 2019). During stressful performance challenges, metacognitive capacity is particularly valuable, helping individuals apply newly learned skills—including mindfulness meditation practice.

In future, more mindfulness at work research should embrace a multi-level approach, investigating cross-level relationships between individual and collective mindfulness processes. Multi-level research in organizations is much needed yet rare (Rafferty et al., 2013). The multi-level nature of workplace mindfulness is important, because scholars have attempted to theorize on the cross-level benefits of individual mindfulness practice (e.g., Good et al., 2016). However, it is logically challenging to simply aggregate individual-level mindfulness practices into a higher level of analysis. This is because mindfulness is unlikely to manifest in the same way across every individual in any given group at any point in time. Similarly to other work concepts such as team performance, the performance of any work team cannot be ascertained by simply compiling individual performance contributions; some individuals contribute more than others at different points in time (Kozlowski and Klein, 2000).

Specific follow-up research opportunities include:

- What sequence of individual and collective mindfulness practices activate which other mindfulness mechanisms? Which dampens others? Which ones interact, which operate independently of each other?
- Which of the five collective mindfulness processes are more, or less, predictive for creating a collective foundation for mindfulness and stress resilience in work teams? How is each linked to individual mindfulness capacity?
- What dosage is necessary of individual and collective mindfulness training, and how much of either is sufficient to embed mindfulness in an organization?
- Can collective mindfulness be trained without individual mindfulness training at all, i.e., exclusively *from the outside in*?

### Mindfulness Training Beyond Meditation Extends Its Transformative Potential

Interdependence theory suggests that most people do not think and act in neutral contexts. Today’s interdependent world looks and sounds different from the monastic backdrop in which the venerable wisdom traditions were forged. They are the foundation for the current interest in mindfulness, yet more context-sensitive training approaches are needed for generating transformative capacity to help release human suffering and encourage flourishing in workplaces (Rupprecht et al., 2019; Selart et al., 2020).

Recall that the Dalai Lama emphasizes the need to complement “closed-eyes” meditative practice with an “eyes-open” focus on compassionate attitudes and behavior, to generate happiness in self and in the world (Dalai Lama, 2005).

Leading mindfulness meditation scholars argue that mindfulness is an umbrella term that describes a large number of processes and practices related to awareness, attention, and acceptance (Creswell, 2017; Van Dam et al., 2017). In addition, mindful organizing experts stress that non-meditative practices complement meditation in generating mindfulness in organizations (Sutcliffe et al., 2016; Reina and Kudesia, 2020). Nonetheless, the terms meditation and mindfulness are routinely used interchangeably in seminal mindfulness intervention publications (see Creswell, 2017; Van Dam et al., 2017). This indicates that the *practice* of meditation is conflated with mindfulness as an *outcome* of a possibly infinite number of “skillful means” (Kabat-Zinn, 2011, p. 3) to bring healing to individuals and society.

The empirical work presented here suggests that workplace mindfulness training that steps beyond a focus on individual meditation to target stress reduction may hold greater potential in transforming stress management capacity, especially at collective levels.

This goes against the notion that the transformative potential of mindfulness may only be unleashed through first-person experience of mindfulness meditation guided and nurtured *via* the second-person perspective of a highly skilled mindfulness trainer (Kabat-Zinn, 2011).

There are at least three reasons why moving beyond a meditation focus might extend the transformative potential of mindfulness interventions for workplaces, as outlined below.

First, in the present study, the second-person perspective provided by the mindfulness trainers in the TMT program included context-sensitive learning facilitation in mindfulness that went beyond teaching meditation. In addition, the training included peer-to-peer learning. The qualitative evidence reported herein for TMT generating mindfulness-based collective stress management skills is in line with recent evidence from similar high-stakes contexts in which mindfulness training was successfully provided by trainers who were domain experts yet had no significant meditation teaching expertise (Jha et al., 2020). This means context awareness and domain expertise may be more important than previously assumed, to render workplace mindfulness training fit for purpose.

Second, the present exploration prompts a re-examination of the axiomatic assumption that mindfulness should be cultivated predominately *via* meditation, and that meditation necessarily produces motivational states that stretch beyond an interest in personal stress reduction, for example relating to collective mindfulness (c.f. Choi et al., 2021). For example, recent research reports indicate counterintuitive effects of mindfulness meditation interventions on work-related outcomes: for example, lower work motivation after 15 min of mindfulness meditation (Hafenbrack and Vohs, 2018); no increase in critical thinking performance after 6 weeks use of the Headspace™ App (Noone and Hogan, 2018); and conflicting evidence on the effect of mindfulness meditation on prosocial motivation (Hafenbrack et al., 2020, 2021).

A close examination of the link between mindfulness meditation and prosociality may shed light on this phenomenon. While most of today’s evidence-based mindfulness interventions are self-focused, intent on calming one’s mind and taking on the stance of a nonjudgmental observer of one’s thoughts and feelings, mindfulness scholars share a widespread assumption that mindfulness training cultivates beneficial outcomes not only for the self but also for others (see Schindler and Friese, 2021 for a review of this evidence). Indeed, recent meta-analyses report significant links between mindfulness and prosocial outcomes (Donald et al., 2019; Berry et al., 2020). However, the same research reviews also report publication bias and low probability of replicability. More pertinently, the meta-analysis of Berry et al. (2020) distinguishes between attitudes of compassionate, empathic concern, and actual prosocial behavior when such behavior would entail costs to the person providing prosocial support (knowledge sharing with a fellow worker, sharing one’s home with a refugee, and so on), and found no reliable effect of mindfulness meditation for the latter.

This makes sense when considering that the target state of mindfulness training as self-regulation of attention (the afore-mentioned operational definition of meditation; Goleman and Schwartz, 1976) is being open and receptive, not motivated to engage in action (Ryan et al., 2021). In fact, the effect of mindfulness meditation on prosociality is moderated by how *independent* or *interdependent* individuals see themselves: for those with independent self-construals, its effect is to decrease prosocial behavior (Poulin et al., in press). This may help explain other recent research reports of mindfulness meditation dampening prosociality (Schindler et al., 2019; Hafenbrack et al., 2021).

Finally, scholars call attention to the adverse effects of mindfulness meditation especially in contexts of latent trauma, urging for a deeper understanding of potential harmful effects of meditation (Van Dam et al., 2017; Baer et al., 2019). Polyvagal theory (Porges, 2011) may help explain why someone who has (consciously or unconsciously) been exposed to traumatic stress in the past may not benefit from prolonged silent meditation practice: it can be experienced as immobilization, the body’s automatic response to overwhelming trauma, prompting a “freeze” response. As a result, the experience may be unpleasant or even cause harm. By the same token, Porges’ (2011) theory also explains why a stronger focus on social engagement in mindfulness training for high-stress work populations (for example individuals serving in the military or working in other contexts where they may experience extreme stress or sustained work pressure) is an alternative antidote to stress at work—and potentially more effective: social engagement between humans who trust each other automatically calms people down.

Clearly, mindfulness-based intervention science needs to balance the need to maintain fidelity to the overall intent of mindfulness to transform suffering in the world on one hand with creating innovative approaches to advancing its scope in society on the other (Kabat-Zinn, 2011). However, a re-examination of the *de-facto* standard in mindfulness training may be timely. In this context, a re-assessment of the 8-week training duration may also be warranted, an arbitrary training timeframe for work populations, and perhaps too short for significant collective-level benefits to develop, as the study’s empirical data suggest.

Specific follow-up research opportunities include:

- To what extent is mindfulness meditation practice an essential ingredient of a workplace mindfulness-based program? How does meditation compare in effectiveness to other, more prosocially oriented mindfulness practices?
- What is the potential and what are potential pitfalls of alternatives to the traditional student-teacher relationship in mindfulness training? What is the role of peer learning and of communities of practice in this?
- What training timeframes are appropriate for workplace mindfulness training? What different timeframe “anchors” beyond the 8-week format have utility? What (blend of) delivery formats is most beneficial for whom?
- When is which type of mindfulness training harmful, for whom, and under what circumstances?

### Limitations

Several limitations pose threats to the validity of the empirical results presented in this study. First, combining individual mindfulness training, traditionally operationalized as an 8-week training program, with the concept of collective mindfulness and its five hallmark processes in equal proportion was a pragmatic choice, rather than a reflection that these two constructs are theoretically equivalent. While this is arguably an important first step in extending the scope and potential of mindfulness training, more careful follow-up examinations are needed to determine which construct or process relates to which other (sub-)mindfulness process and in what way. Second, quantitative assessments lacked a comprehensive measure of individual mindfulness. Third, an uneven number of individuals participated in Study 2’s two-group qualitative assessment. Fourth, an uneven number of females and males participated in the quantitative evaluation and both genders were analyzed together. Fifth, the same author who developed and delivered the training also evaluated the data collected about its impact. Finally, only one specific combination of individual and collective mindfulness was examined in this pilot; this provides an incomplete theoretical picture of the potential that “next-generation” mindfulness training might (or might not) represent for individuals at work, especially those under intense constant pressure and scrutiny, as in the military. This is certainly but the first step towards a more comprehensive understanding of the transformative potential of mindfulness training in organizations.

## Conclusion

For several years, mindfulness scholars have argued that workplace mindfulness research should embrace a multi-level approach, investigating cross-level relationships between individual and collective mindfulness literatures (Sutcliffe et al., 2016; Reb et al., 2020). The present study heeds this call by creating a cross-level pilot mindfulness intervention entitled TMT. This innovative mindfulness training program combines essential ingredients of a “traditional” individually focused MBI with the hallmarks of collective mindfulness. TMT was trialed with two high-stress military populations operating in a context in which dedication and self-sacrifice are prized values, and a public perception of meditation-oriented “mindfulness as stress relief” (Choi et al., 2020) may be counter-cultural, while the idea of *mindfulness as team sport* may be more fit for purpose. Hence, this was deemed a suitable setting to explore new and yet untapped ways in which mindfulness training may help cultivate “next-generation” transformative inner qualities, for the benefit of the individual as well as for all.

The study’s empirical investigations indicate that TMT appears no less effective in generating individual stress management capacity than a “traditional” MBI, and it seems to show more potential for cultivating collective stress management skills. In addition, individuals’ ability to apply their newly learned mindfulness meditation skills to stressful work situations may depend on the development of a collectively mindful team culture—in other words, individual and collective mindfulness development may be interdependent.

Drawing on interdependence theory to discuss these findings, the paper proposes that mindfulness intervention science and practice should apply a process-based approach (Hofmann and Hayes, 2019) to help extend the transformative potential of mindfulness training for workplaces, and ultimately in society. In particular, “next-generation” workplace mindfulness research should apply a multi-level approach to reflect the multi-level nature of mindfulness in organizations, enacted in non-meditative processes and social engagement as much as through meditative practice (Sutcliffe et al., 2016).

Concretely, this means combining and comparing meditative with non-meditative mindfulness practices and including relevant elements from individual and collective mindfulness in training design and delivery, to examine their respective impact for individuals and teams at work. The present study especially recommends that scholars should move on from an exclusive focus on meditation as primary tool of mindfulness training and explore potentially untapped benefits of “eyes-open” mindfulness practices in workplaces. This may be particularly relevant in high-stress populations for whom meditation-focused mindfulness training may not always be most fit for purpose.

The ultimate aim of this work is to respectfully prompt a shift in focus for workplace mindfulness intervention science, away from defaulting to 8-week mindfulness meditation training to help participants manage stress by themselves, and towards a sense that people at work are interdependent, that they have each other’s’ back, and that stress management may be more of a collective responsibility rather than something that needs to be shouldered by individuals in isolation of others. Such a focal shift loops back to the altruistic aspiration of mindfulness in Eastern contemplative traditions.

In the words of the Dalai Lama; “(y)ou should not be content with working for your own personal benefit alone” (2005, 94). Cultivating capability to understand and overcome suffering and conflict, for one and all, is what the transformative potential of mindfulness is about.

## Data Availability Statement

Descriptive statistics for the study are included in the article/Supplementary Material, further inquiries can be directed to the corresponding author.

## Ethics Statement

The studies involving human participants were reviewed by the United Kingdom Ministry of Defence Research Ethics Committee (MODREC) who gave a favorable opinion, and ethical approval to conduct this research was also obtained from the university ethics committee of the Cranfield University. The participants provided their written informed consent to participate in this study.

## Author Contributions

JTM led the design, delivery, analysis, interpretation, and writeup of the study. AC served as Principal Investigator and contributed to the design of the study and ethical approval process, led the data collection of Study 2, and provided overall research guidance. DQ conducted the quantitative data analysis, co-created the qualitative data analysis for Study 2, and provided guidance on data interpretation. All authors contributed to manuscript revisions, read and approved the final submitted version.

## Funding

The empirical field research for this study was funded by the United Kingdom Ministry of Defence, under the Strategic Edge Through People (SETP)2040 programme, reference number TIN 2.068 Task 7 Leveraging Workplace Mindfulness for Transformative Change, contract number DSTLX-1000069524.

## Conflict of Interest

The authors declare that the research was conducted in the absence of any commercial or financial relationships that could be construed as a potential conflict of interest.

## Publisher’s Note

All claims expressed in this article are solely those of the authors and do not necessarily represent those of their affiliated organizations, or those of the publisher, the editors and the reviewers. Any product that may be evaluated in this article, or claim that may be made by its manufacturer, is not guaranteed or endorsed by the publisher.
