# Supplementary material for: Extending the Transformative Potential of Mindfulness Through Team Mindfulness Training, Integrating Individual With Collective Mindfulness, in a High-Stress Military Setting
Source: Front Psychol. 2022 Jun 30;13:867110. doi: 10.3389/fpsyg.2022.867110 (PMC9282162; doi:10.3389/fpsyg.2022.867110)
Supplement: Supplementary file 1 [file Data_Sheet_1.docx]

Supplementary Material

# Supplementary Tables

Table A: Working memory test descriptive statistics

| **Training condition** | **N (observations)** | | | **Mean score** | | | **Standard deviation** | | |
| --- | --- | --- | --- | --- | --- | --- | --- | --- | --- |
|  | Time points | | | Time points | | | Time points | | |
|  | T1 | T2 | T3 | T1 | T2 | T3 | T1 | T2 | T3 |
| Individual | 51 | 45 | 34 | 46.51 | 48.93 | 42.26 | 15.47 | 19.21 | 20.22 |
| Team | 49 | 37 | 31 | 42.98 | 46.57 | 45.45 | 15.88 | 20.88 | 18.30 |

Table B: Individual resilience scale descriptive statistics

| **Training condition** | **N (observations)** | | | **Mean score** | | | **Standard deviation** | | |
| --- | --- | --- | --- | --- | --- | --- | --- | --- | --- |
|  | Time points | | | Time points | | | Time points | | |
|  | T1 | T2 | T3 | T1 | T2 | T3 | T1 | T2 | T3 |
| Individual | 53 | 47 | 34 | 4 | 4.22 | 4.16 | .37 | .42 | .37 |
| Team | 49 | 36 | 31 | 3.99 | 4.21 | 4.09 | .56 | .42 | .47 |

Table C: Mindful organizing scale descriptive statistics

| **Training condition** | **N (observations)** | | | **Mean score** | | | **Standard deviation** | | |
| --- | --- | --- | --- | --- | --- | --- | --- | --- | --- |
|  | Time points | | | Time points | | | Time points | | |
|  | T1 | T2 | T3 | T1 | T2 | T3 | T1 | T2 | T3 |
| Individual | 53 | 47 | 34 | 5.23 | 5.17 | 5.06 | .56 | .81 | .79 |
| Team | 49 | 36 | 31 | 5.20 | 5.36 | 5.31 | .65 | .55 |  |
